# The Need for Earlier Implementation of Comprehensive Sexual Education Within a Formal Classroom Setting and Beyond Based on the Influences of Technology

**DOI:** 10.7759/cureus.28552

**Published:** 2022-08-29

**Authors:** Alexandria L Betit, Christina Kennedy

**Affiliations:** 1 Physiology, Alabama College of Osteopathic Medicine, Dothan, USA

**Keywords:** young adolescents, age-appropriate sexual education, abstinence only education, social media, comprehensive sexual education

## Abstract

Abstinence-only education taught predominately via formal classroom instruction has continuously been emphasized throughout history and in classrooms today. Although abstinence-only methods are often stressed, contraceptive education is occasionally but not consistently implemented in school curricula. A variety of other instructional delivery methods include student-peer education, education outside of the formal classroom setting, community youth service programs, education via telehealth, educational videos, self-study websites and social media. Providing comprehensive sexual education utilizing multiple instructional delivery methods could close the gap in sexual education for adolescents.

The age at which sexual education instruction is introduced has remained relatively unchanged throughout history. Adolescents are being formally educated within classrooms as early as grade five, although they are often exposed to informal and potentially misleading information regarding sexual education much earlier than this. In part, this is due to the relatively recent emergence and subsequent influence of technology such as social media. Thus, given the influence of technology such as social media in recent history we need to reevaluate the age of formal sexual education and increase comprehensive sexual education resources.

Additionally, it is important to note that sexual education instruction provided solely in formal classroom settings may not provide sufficient information for youth to make informed decisions. Thus, sexual education information including abstinence and contraceptive methods should be provided through additional means via utilizing differing instructional delivery methods in conjunction with formal classroom instruction. For example, comprehensive sexual education should also be provided in healthcare offices including pediatric and obstetrics and gynecology (OBGYN) offices. Sexual education could include discussing/providing external resources such as pamphlets that incorporate social media and other links to online resources that provide a more inclusive, accurate educational experience within a safe environment. This would allow healthcare professionals to provide a better targeted and engaging educational experience to adolescents as well as proactively allow for exposure of younger adolescents to helpful educational resources.

## Introduction and background

Sexual education has covered a broad array of topics including anatomy and physiology, consent and healthy relationships, sexually transmitted diseases (STDs), and pregnancy prevention. However, as of March 1, 2022 only 29 states and Washington, DC, mandate sexual education in schools [1].

Historically, many sexual education programs were conducted formally within school classrooms with an emphasis placed on abstinence-based instruction. In 1996, in order to receive federal funding, the school needed to teach abstinence-only methods as the expected standard for adolescents [2]. However, a 2004 report reviewing abstinence-only education detailed that under the Special Projects of Regional and National Significance (SPRANS) Community-Based Abstinence Education Project Grants, abstinence-only programs were solely allowed to place emphasis on the ineffectiveness of contraceptive methods rather than mentioning them as potential approaches to prevent pregnancy [3]. Nationally, from 1988 to 1999 the United States (U.S.) saw an increase in the number of teachers who focused on abstinence-only education [4]. In 1999, although the effectiveness of contraception was mentioned, 23% of sexual education teachers emphasized abstinence-only methods compared to 2% of teachers in 1988 [4]. In 1998, a national survey was completed by 825 public school representatives which illustrated that 23% of all U.S. school districts focused only on abstinence and if they did mention contraception, it was solely to acknowledge their limitations [5]. Of these public schools, 51% taught abstinence as the preferred method, although they allowed instruction on the effectiveness of contraception [5]. This national survey also illustrated that public schools within the South were nearly five times more likely to have abstinence-only policies when compared to schools within the Northeast [5]. Furthermore, in 1999, over 600 fifth- and sixth-grade sexual education teachers in U.S. public schools were surveyed and over half taught abstinence as the only method of protection against pregnancy [6].

This emphasis on abstinence-only methods has continued to persist today in more recent curriculums. From 2001 to 2008 the government allotted a progressively increasing amount of money to fund abstinence-only educational methods [2]. Proportionally, utilizing national data, it was determined that the number of students aged 15 to 19 who received abstinence-only education increased from 1995 to 2002 [7]. As demonstrated by a nationally representative study of data from the National Surveys of Family Growth from 2006 to 2013, females and males aged 15 to 19 years old reported an increase in formal instruction on saying no to sex without instruction on contraceptive methods (22% to 28% for females and 29% to 35% for males) [8]. As of March 1, 2022, 20 states require information regarding contraception and 39 require information on abstinence, 28 of which require that abstinence be stressed [1].

Strictly focusing on formal education curriculums, excluding parental roles, abstinence-only education curriculums have not been shown to reduce teen pregnancy or sexual activity and are not as informative as comprehensive curriculums. There are some abstinence-based models being implemented in some states that are also providing instruction on contraceptive methods. Yet, the way in which these educational topics are being presented to youth as early as grade five varies [6]. Some instructional methods such as formal education in the classroom have historically been preferred, but more recently with the growing usage of technology, other instructional methods have emerged such as educational videos, self-study websites and social media.

## Review

As of March 1, 2022, although 28 out of the 39 states that include abstinence instruction stress abstinence methods, 12 of those 28 states are providing contraceptive education methods as well [1]. Further, when sexual education instruction is provided, 20 states require that it must include contraception education [1]. Ultimately, regardless of religion or race, less than half of states are requiring comprehensive sexual education instruction [9-11].

Instructional delivery methods

Historically, sexual education has been taught predominately via formal didactic education within a classroom setting. Although sexual education is still taught formally within classrooms today, over time other methods of instructional delivery have become more popular outside of the classroom. Some of these methods include student-peer education, education outside of the formal classroom setting, community youth service (CYS) programs, education via telehealth, educational videos, self-study websites and social media.

One instructional method utilized includes student-peer education. Student-peer education involves students teaching other peers with a similar background to their own. This has been utilized as an effective method throughout history to educate young members of society on differing topics. As it relates to contraception, the Birth Control Handbook was utilized largely from 1968 to 1975 as a means of student-peer education throughout Canada [12]. The text was created by a group of university students targeting peers with emphasis placed on contraception methods and feminism [12]. Another ongoing student-peer education intervention implemented in six schools in Scotland targeting adolescents 14 to 16 years old includes the Sexually Transmitted infections And Sexual Health (STASH) program [13,14]. This intervention utilized trained peers to, “spread positive sexual health messages,” via in-person conversations with peers as well as on social media websites such as Facebook [13,14].

Some research suggests providing education outside of a formal classroom setting may be utilized as an appropriate means to educate youth on sexual health matters. For example, a suggested approach regarding contraception education includes utilizing non-school-based settings such as mall-based clinics given students’ preference for instruction outside of the classroom [15].

Another approach to education involves employing CYS programs. CYS programs are aimed at fostering youth participation within the community in an effort to influence their health outcomes [10]. Health clinics, senior citizen centers, nursing homes and child day care centers were all places within the community in which CYS programs were established with the goal of decreasing sexual behaviors in youth [10]. In this study, youth were exposed to a classroom curriculum accompanied by 3 hours per week of service-learning education [10]. The results of the study indicated that CYS programs can be beneficial in reducing unprotected sex, STDs and unintended pregnancy compared to controls [10]. For example, eighth graders’ unprotected sex rates increased within the control groups and decreased by 30.8% for sex without a condom and 27.7% for sex without contraception within the groups who received education through the CYS program [10].

Telehealth has also been crucial in providing increased access to sexual health education. Healthcare providers are in a pivotal position as they can provide accurate appropriate educational resources concerning sexual health to adolescents. More recently as barriers to health are being recognized, such as insufficient access to healthcare due to regional barriers, healthcare providers have turned to more efficient outreach tactics such as telehealth. One study trialed utilizing telehealth services to educate 16-year-old high school female students within a rural area in West Virginia on topics such as contraception and STDs [16]. A follow-up study including males was then performed a year later which showed that telehealth is valuable and beneficial in teaching sexual education to teenagers within rural areas [17]. The results of this study indicated that the number of sexually active adolescents, “always using condoms,” and, “always using birth control,” increased from 46.2% to 58.8% and 35.3% to 64.7%, respectively [17].

Amid the implementation of technology, other educational methods have gained popularity such as educational videos. In general, videos have been utilized for decades and have been shown to be an effective educational tool. In 1978 in Canada, an educational film, “It Couldn’t Happen to Me,” targeting teenagers were shown to members of a sexual education workshop including high school and university students [18]. The film sought to expose reasons why teens were not utilizing contraception in efforts to enhance the effectiveness of contraception education programs [18]. Another educational video intervention, Plan A, targeted teenagers aged 16 to 19 years old [11]. The educational video largely emphasized the effectiveness of long-acting reversible contraceptives (LARCs) including intrauterine devices (IUDs) and the implant [11].

Furthermore, society has seen an increase in internet usage leading to greater popularity of virtual methods of instruction. Such methods include self-study websites as well as the influences of social media. One example of a self-study website includes Contraception Choices which provides information about physiology and contraception [19]. The website examines various contraceptive methods in an effort to aid women in determining the benefits and cons of each method as well as the best method for them [19]. Information is presented utilizing interactive features such as videos, pictures, and tables. This website also helps inform and educate women about the different types of contraception that are available so they may be more prepared when speaking with their healthcare providers [19]. Another self-study resource includes a website designed by a board-certified obstetrician-gynecologist (OBGYN) initially tested by OBGYN resident physicians and undergraduate students from West Virginia with the goal of educating high school adolescents [20]. The trialed website includes a series of short videos totaling 30 minutes detailing different topics such as contraception, physiology, and STDs [20].

Social media has played an influential role in sexual education implementation as well (Table 1). Social media has advantages including its easy accessibility and expansive influence. However, there are also disadvantages associated with social media including potential difficulties screening for inaccurate information as well as the inability to preclude certain facets of information until an age-appropriate time. This can be particularly problematic given that social media platforms, including TikTok and Facebook, cover information concerning sexual health and contraception methods. Thus, social media is being utilized by adolescents as an educational reference [21]. One analysis involved the creation of a TikTok account for a hypothetical 15-year-old to determine the videos that came across a typical 15-year-old’s timeline based on the entry of hashtags including #sexeducation and #healthclass [21]. Results indicated the presence of a wide variety of sexual health information including topics such as female anatomy, sexual pleasure, and contraception which specifically totaled 13% of the videos reviewed [21]. In evaluating the effectiveness of Facebook utilization as a supplement to traditional healthcare counseling concerning contraception, one study concluded that Facebook is not only an appropriate means of providing contraception education but should be encouraged in conjunction with traditional physician guidance [22]. Overall, the information provided on social media is readily available to youth with access to technology, potentially prior to formal classroom sexual education instruction which could be problematic.

**Table 1:** Instructional delivery methods Multiple forms of sexual education delivery methods and the targeted age groups. Some self-study websites provide sexual education to undergraduate college students [20] and those between 15 and 30 years old [19]. Student-peer education has provided sexual education to university students [12] and those between 14 and 16 years old [13]. Educational videos have provided sexual education to those between 16 and 19 years old [11] as well as high school students and university students [18]. Social media provides sexual education information to all ages utilizing TikTok [21] as well as those between 18 and 45 years old utilizing Facebook [22]. Healthcare providers provide sexual education to high schoolers around 16 years old via usage of telehealth [16]. Sexual education information has been addressed outside of a school setting such as in malls targeting those between 14 and 20 years old [15]. Community Youth Service programs have targeted those within seventh and eighth grade [10]. Formal sexual education within the classroom is being taught to those in fifth through 12th grade [4-9].

Instructional Delivery Method	Age Group Targeted
Self-Study Website	Undergraduate College Students, 15 to 30 years old
Student-Peer Education	14 to 16 years old, University Students
Educational Video	16-19 years old, High School Students and University Students
Social Media	All ages utilizing TikTok, ages 18 to 45 on Facebook
Healthcare Providers -Telehealth	High Schoolers (16 years old)
Outside of the School Setting (i.e. malls)	14 to 20 years old
Community Youth Service Programs (Community Involvement)	Seventh and Eighth grade
Formal Education Within the Classroom	Fifth through 12th grade; Middle and High Schoolers

Age of initiation of sexual education

The age/grade level of initiation of sexual education programs throughout the U.S. middle/high schools has not markedly changed over the course of history despite the advancement of technology and the plethora of information that accompanies this time progression. As early as 1968 through student-peer education, university students were provided contraceptive education with the creation of the Birth Control Handbook 1968, primarily lasting from 1968 to 1975 [12]. From 1988 to 1999, even though abstinence education was stressed, contraception and condom usage were taught within a classroom setting at some point to adolescents throughout grades seven through 12 [4]. In 1998, adolescents in grades sixth through 12th grade were exposed to sexual health education [5]. Additionally in 1998, a national survey of sexual health programs in grades six through 12 was conducted and illustrated that 14% of schools addressed abstinence and contraceptive education and 51% of school districts stressed abstinence education, but the effectiveness of contraception was mentioned [5]. In 1999, as supported by results from a national representative survey, sexual education, although largely focused on abstinence education, was being taught to fifth-grade students [6]. Overall, within the late 1900s, the age of initiation of formal sexual education did not drastically change with the earliest age of initiation being in the fifth grade.

More recently, in 2015 a study involved the implementation of a federally funded, community-based abstinence education program delivered by a nursing center targeting Philadelphia youth aged 12 to 18 years old [23]. 12-year-olds are either in sixth or seventh grade depending on their birthday. Furthermore, many state-required sexual education plans are largely non-specific in relation to the age at which sex education is implemented. Multiple states’ plans mention initiating sexual education programs at an “age-appropriate,” time [24]. On the other hand, some states are more definitive regarding the time of exposure to sexual health education. For example, as of 2019, according to Illinois state laws regarding sexual education, if offered, contraception and abstinence education will be provided in grades six through 12 [24]. Additionally, North Carolina state laws mention that each local school must provide sexual education starting within the seventh grade [24]. In the Centers for Disease Control and Prevention’s (CDC) Health Education Curriculum Analysis Tool (HECAT) in 2012, it was recommended to include education regarding contraception in the eighth grade [25]. Overall, this information illustrates the unchanging nature of the age of implementation of sexual education across history (Table 2). However, this may be problematic considering the early exposure adolescents have to various social media platforms which often include information regarding sexual health.

**Table 2:** Grade of initiation of sexual education across history Content included within sexual education programs across history and the grade level in which these programs were being taught. From 1968 to 1975, university students were given information regarding contraceptive education [12]. From 1988 to 1999 those between grades five and 12 were given information concerning contraception, although abstinence education was stressed [4-6]. In 2012, the CDC recommended that contraceptive education be provided within the eighth grade [25]. In 2015, abstinence education was taught to students in sixth or seventh grade through 12th grade [23]. In 2019, both contraception and abstinence education was provided to students in grades six through 12 in Illinois and to students in grades seven through 12th grade in North Carolina [24].

Year(s) of Instruction	Grade Level	Instructional Methods
1968-1975	University Students	Contraceptive education
1988-1999	Seventh through 12th grade	Abstinence education was stressed, but contraceptive education was provided
1998	Sixth through 12th grade	Abstinence education was stressed, but contraceptive education was provided
1999	Fifth grade	Abstinence education was stressed, but contraceptive education was provided
2012	Eighth grade	CDC recommends contraception education
2015	Sixth/Seventh through 12th grade	Abstinence education
2019	Sixth (Illinois) or Seventh (North Carolina) through 12th grade	Contraception and abstinence education

In this new age of technology, based on a study in 2016 the average age adolescents get their first phone is around 10 years old [26]. Moreover, 11% of adolescents started their first social media account younger than 10 years old and 39% started their account between 10 and 12 years old [26]. Comparatively, this is younger than schools that provide formal sexual education.

Social media platforms often include videos addressing sexual education topics such as contraception, female anatomy, sexual pleasure and sexual health [21]. Yet, these topics may be inconsistent and/or contradictory to formal instruction adolescents receive within classrooms [21]. Moreover, as social media exposes adolescents to sexual education it potentially acts as an adjunctive resource and/or a replacement for traditional formal sexual education within classrooms [21]. This may partially be due to social media platforms creating a more comfortable and encouraging environment compared to classrooms as adolescents are able to explore their sexuality privately [21]. However, as there is often no screening for accuracy or misinformation regarding these sexual health topics on social media platforms [21], adolescents may be exposed to incorrect sexual health information prior to formal instruction in school. All in all, due to younger adolescents having access to technology such as social media platforms, they are being exposed to information regarding sexual activity and contraceptives at a much earlier age than in the past and this information may not be factual. Therefore, it is imperative we start exposing our younger members of society to sexual education instruction in some manner before the current age of initial exposure.

As some states' sexual education policies mention, it is often suggested that sex education be provided at an age-appropriate time. Yet, the meaning of this term is relatively unclear. A survey of over 600 U.S. public school fifth and sixth-grade sexual education teachers revealed that many recommend education regarding contraception usage at grade six or earlier [6]. Further, some research suggests sexual education instruction should be initiated as early as fourth grade. One reason for this is that these young adolescents are undergoing puberty which raises questions about sexuality and fertility [27]. Particularly, an article from Georgetown University researchers suggests that sexual health instruction should begin as early as age 10 even though most current programs focus solely on older adolescents [27]. By considering only older adolescents, this ultimately fails younger adolescents (between 10 and 14 years old) and forces them to gather information from other sources including the media [27]. Another source from The Hospital for Sick Children states that adolescents aged nine to 12 should be exposed to contraception and safer sex methods [28]. Ultimately, even though access to technology has changed over the years, the age at which sexual education instruction is initiated has not appropriately changed in proportion.

Overall, the U.S. is still seeing gaps in contraception education in many states, and this has not significantly changed from earlier in history. It is critical that more schools focus on comprehensive sexual education programs that include contraception given they have more benefits including positively influencing pregnancy rates and overall sexual health decisions when compared to abstinence-only education programs [2,29].

Abstinence-only vs comprehensive sexual education programs

Many schools utilize the abstinence-only delivery method. This is problematic because abstinence-only education is not effective in delaying sexual activity or decreasing the risk of teen pregnancy [2]. However, more comprehensive sexual education programs that included contraceptive education were associated with lower pregnancy rates and a “decreased likelihood of a teen becoming sexually active” [2]. Further, government-funded abstinence-only education programs which include virginity-pledge programs have been shown to be ineffective as displayed in a national longitudinal representative study of teens from 1995 to 2001 [30]. This study indicates that those who pledged to pursue abstinence had comparable rates of sexual activity to those who did not make an abstinence pledge [30]. Additionally, it was discovered that those who had pledged to remain abstinent utilized contraceptive methods less frequently than non-pledgers [30].

Moreover, abstinence-only education programs are not always reliable. In a report from the United States House of Representatives Committee on Government Reform-Minority Staff Special Investigations Division in December of 2004, it was stated that abstinence-only curricula contain inaccurate and misleading information about risks associated with sexual activity as well as incorrect information regarding contraception effectiveness [3]. In one literature review citing research from 2010 to 2015, it was determined that abstinence-only programs were not solitarily effective in reducing sexually transmitted infections (STIs) or pregnancy [9]. Therefore, given the unreliability of abstinence-only education policies, a more consistent and comprehensive level of instruction which includes contraception education should be instated across the U.S. This should be done at an earlier age, given the consequences of new technology/the internet resulting in earlier exposure of adolescents to potentially incongruent sexual health messages on social media.

## Conclusions

Abstinence-only education has been taught predominately via formal classroom instruction and has continuously been emphasized throughout history. Abstinence-only education has continued to be taught within classrooms today despite its stated relative ineffectiveness, although sexual education is not consistent between states. For example, even though abstinence-only methods are often stressed, contraceptive education is occasionally implemented in some schools’ curricula. However, a variety of other instructional delivery methods for sexual education are also utilized outside of the classroom. Yet, the age at which sexual education instruction is introduced has remained relatively unchanged. Given the influence of technology such as social media in recent history, we need to reevaluate the appropriate age of implementation of sexual education. Comprehensive sexual education needs to be available to youth at an earlier age prior to the aforementioned earliest age of implementation within the fifth grade. We need to ensure adolescents are provided with comprehensive sexual education and prevention resources prior to exposure to potentially misleading information found within the media.

Collectively, to provide a more complete sexual education to youth, differing instructional delivery methods should be utilized in conjunction with formal classroom instruction. Sexual education taught solely within a formal classroom setting does not provide sufficient resources to youth to make informed decisions especially given the influence of social media. Thus, sexual education information including contraceptive methods should be provided through additional means. For example, comprehensive sexual education should be provided in healthcare offices including pediatric and OBGYN offices in order to provide a more inclusive, accurate educational experience within a safe environment. This can be addressed through discussing/providing sexual education materials including information regarding contraception usage within healthcare provider’s offices through external resources including pamphlets that incorporate social media and/or links to self-study websites. This would allow healthcare professionals to provide a better targeted and engaging educational experience to adolescents who generally religiously interact with technology within a safe environment as well as proactively allow for exposure of younger adolescents to helpful educational resources.
